# Engineered biomimetic micro/nano-materials for tissue regeneration

**DOI:** 10.3389/fbioe.2023.1205792

**Published:** 2023-07-04

**Authors:** Feng Han, Qingchen Meng, En Xie, Kexin Li, Jie Hu, Qianglong Chen, Jiaying Li, Fengxuan Han

**Affiliations:** ^1^ Department of Orthopaedic Surgery, The First Affiliated Hospital, Suzhou Medical College, Orthopedic Institute, Soochow University, Suzhou, Jiangsu, China; ^2^ China Orthopaedic Regenerative Medicine Group (CORMed), Hangzhou, Zhejiang, China

**Keywords:** micro/nano-materials, tissue engineering, repair, regeneration, biomimetic microstructure

## Abstract

The incidence of tissue and organ damage caused by various diseases is increasing worldwide. Tissue engineering is a promising strategy of tackling this problem because of its potential to regenerate or replace damaged tissues and organs. The biochemical and biophysical cues of biomaterials can stimulate and induce biological activities such as cell adhesion, proliferation and differentiation, and ultimately achieve tissue repair and regeneration. Micro/nano materials are a special type of biomaterial that can mimic the microstructure of tissues on a microscopic scale due to its precise construction, further providing scaffolds with specific three-dimensional structures to guide the activities of cells. The study and application of biomimetic micro/nano-materials have greatly promoted the development of tissue engineering. This review aims to provide an overview of the different types of micro/nanomaterials, their preparation methods and their application in tissue regeneration.

## 1 Introduction

Due to lifestyle changes and population aging, the incidence of various diseases, such as degenerative diseases, cancer and trauma, has been increasing. Therefore, there is an increasing demand for tissue and organ transplantation in clinical practice (Bakhshandeh et al., 2017; Nii and Katayama, 2021). However, the repair or replacement of organs and tissues remains a complex and unsolved problem. Current graft materials cannot be widely used due to limitations such as material mismatch and immune rejection (Chung et al., 2017). Tissue engineering which integrates different disciplines, such as life sciences and materials sciences, has a potential for the development of alternative therapeutic strategies to repair damaged tissues and organs.

The basic concepts of tissue engineering include seeding cells on scaffolds of a certain shape, expanding the cells continuously to form a composite system of cells and biomaterials, and finally implanting them into the patient. After *in vivo* implantation, the polymer system will gradually degrade, allowing regeneration of new tissues. In this functional and biomimetic system, the three major elements are the selection of seed cells, the construction of scaffolds, and the regulation of cell-biomaterial interactions (Li et al., 2020). Biomaterials used for tissue engineering must have the following properties: (1) It has good biocompatibility, non-toxic side effects on cells and tissues; (2) The surface of the materials is conducive to cell adhesion and proliferation, and can induce cell growth according to the predetermined morphology; (3) The degradation rate should match the new tissue formation rate; (4) Three-dimensional (3D) structure. Studies have shown that the surface microstructure of biomaterials has a great influence on cell morphology, adhesion, directional growth and bioactivity (Yu et al., 2020; Han et al., 2022).

Biomaterials are natural or artificial materials used to replace or repair tissue. For instance, the annulus fibrosus (AF) has a collagen-rich fibrous lamellar with highly aligned collagen fibers tilted 30° from the horizontal axis. The microstructure allows the spine to resist complex loading. Tendon has a hierarchical organization, and the subunits of the tendon are the fascicles, which consist of highly aligned collagen fibers. With the continuous improvement of tissue engineering and regeneration, plenty of biomaterials have been developed. With the in-depth understanding of the microstructure of different tissues and organs, researchers have noted that it is important to simulate the microstructure of tissues and organs by biomaterials at a more microscopic scale to achieve better tissue repair (Craciunescu et al., 2021). Tissue engineering generally controls the growth and development of cells in three scales. The millimeter scale determines the overall shape of the engineering tissue. The micron scale determines the pore size of the material and regulates cell migration and growth. The nanometer scale determines the physical and chemical properties of material surface, which regulates cell adhesion and gene expression. The properties of extracellular matrix (ECM), such as matrix stiffness, surface topography, and chemical composition, can affect cell behavior spatially and temporally, which is attributed to its fusion with mechanical transduction and molecular recognition (Wang et al., 2018). Those will affect cell phenotype, gene expression, and ultimately cell fate. Moreover, the geometry of ECM also has a great influence on their mechanical properties. It can be seen that the micro/nano structure of ECM has a great impact on regulating cell behavior, which in turn affects tissue organization. Therefore, the preparation of biomaterials with certain micro and nano structures is of great significance for tissue repair and regeneration.

As one of the three elements of tissue engineering, the scaffold is the foundation of constructing engineered tissues. Suitable 3D scaffolds can simulate the microstructure of tissues and provide a suitable microenvironment for cell and tissue growth. Micro/nano-materials have good properties of general biomaterials. The special pores of micro/nano-materials can support nutrient transport, absorption, and tissue growth (Carotenuto et al., 2022). For specific tissues, such as bone and cartilage, higher requirements are also put forward for the mechanical properties of micro/nano-materials. Therefore, more and more attention has also been paid to the mechanical properties. In recent years, a variety of multi-functional micro/nanomaterials have been applied in tissue engineering, exhibiting a broad application prospect. However, the application of micro/nano-materials in tissue engineering is still in the early stages, and there are still many problems regarding the clinical application that need to be solved. The problems include developing suitable micro/nanomaterials to simulate the microstructure of tissue, constructing an ideal cell-material interface to induce directional differentiation of cells, making allogeneic biological tissues and cells cultured on micro/nano scaffolds from immune system recognition and rejection, maintaining the viability and function of cultured cells for a long time, and further improving the biocompatibility of micro/nano-materials.

**FIGURE 1:**
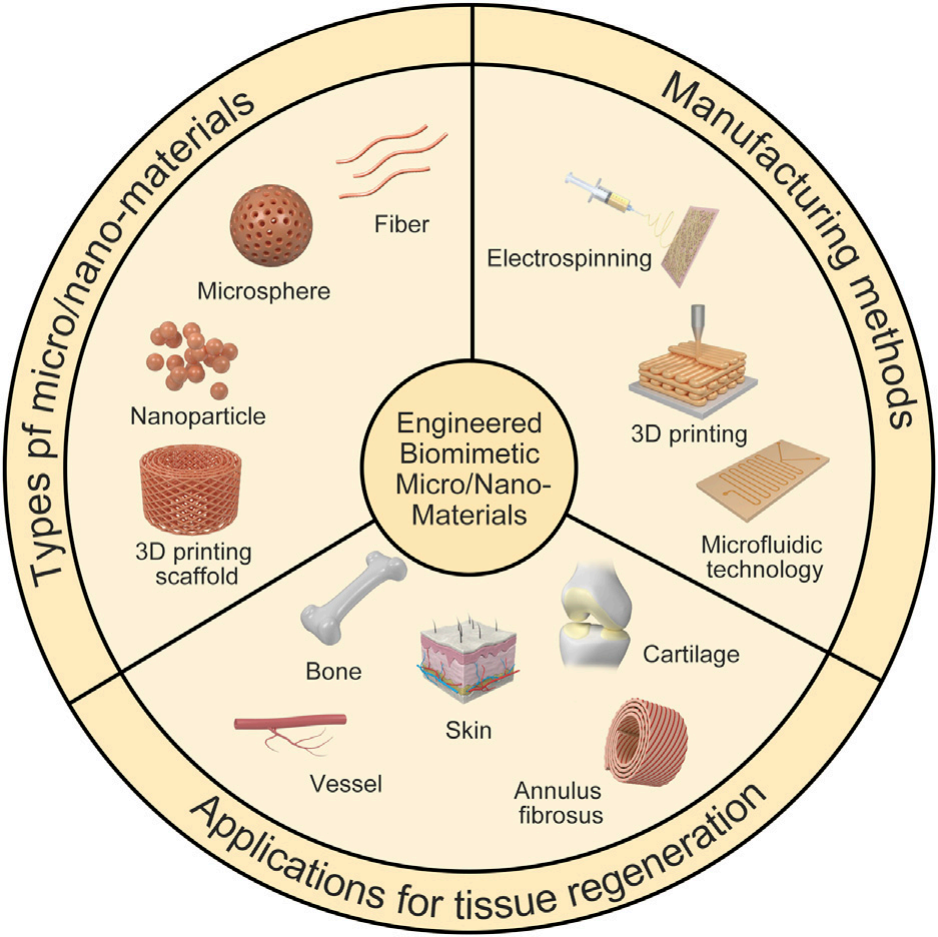
Illustration of engineered biomimetic micro/nano-materials for tissue regeneration. The micro/nano structures including micro/nano fiber, nanoparticle, microsphere and 3D printing scaffold, and the biomedical applications referring to bone repair, AF repair and vascular grafts are shown as examples. Manufacturing methods for different micro/nano-materials are also shown.

Herein, we provide a comprehensive overview of the current research progress on micro/nanomaterials and introduce the types of micro/nanomaterials and their applications (Figure 1). First, the importance of scaffold micro/nanomaterials on cell behavior and biomimetic microstructure is highlighted. Next, several preparation methods of scaffolds are introduced. Then, examples of the application of micro/nanomaterials in bone, cartilage, blood vessels and other tissues are introduced. Finally, some limitations and challenges are briefly discussed.

## 2 Manufacturing methods for micro/nano-materials

### 2.1 Electrospinning

**FIGURE 2:**
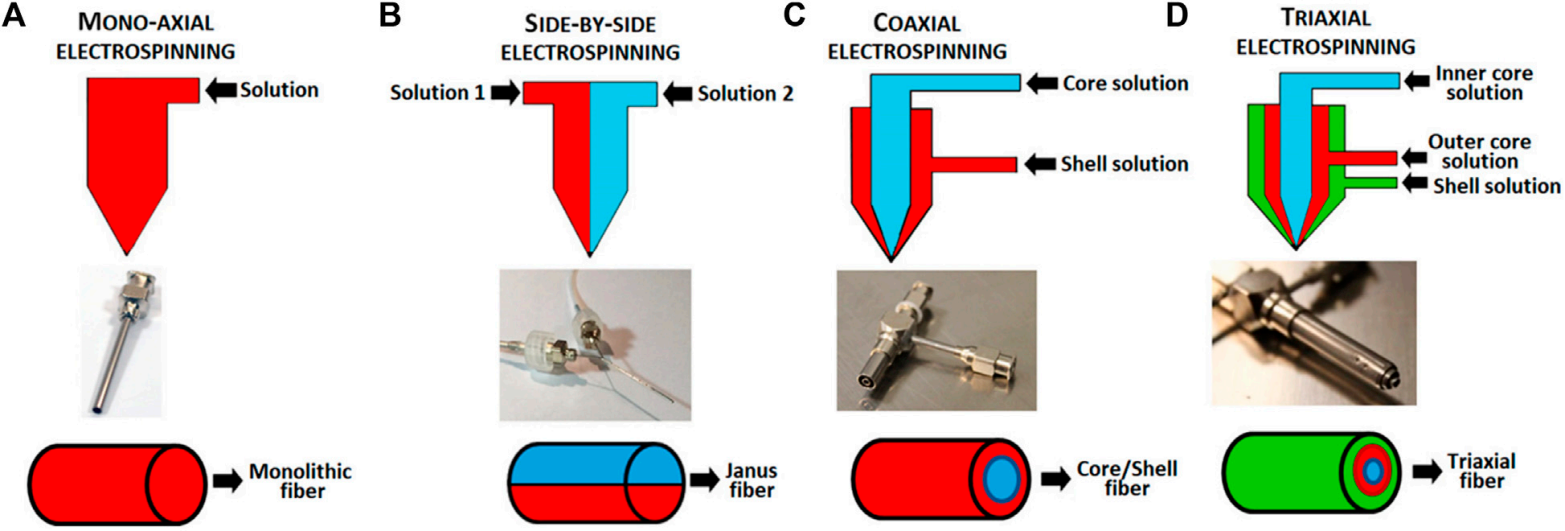
Schematic of electrospinning setup and the fibers output. **(A)** mono-axial electrospinning; **(B)** side-by-side electrospinning; **(C)** coaxial electrospinning and **(D)** triaxial electrospinning (Luraghi et al., 2021).

Electrospinning technology can continuously produce micro/nano-sized ultrafine fibers (Zamani et al., 2018). The scaffold prepared using the electrospinning technology has a unique micro/nano structure and proper mechanical properties (Figure 2). It can simulate the micro/nano-network structure of natural ECM and has unique advantages in the preparation of tissue engineered scaffolds (Recum et al., 1996; Flemming et al., 1999; Mitragotri et al., 2015). Generally, the electrospinning device consists of a high-voltage source, a solution reservoir, and spraying and receiving devices. The electrospinning process can be divided into five processes, including fluid charging, Taylor cone formation, jet stretching and whipping, jet solidification, and fiber reception (Liu et al., 2012; Zamani et al., 2018). The most important process is the formation of the Taylor cone. The electrospinning process can be influenced by many factors such as the solvent properties (e.g., polymer type, concentration, viscosity, conductivity), the processing factors (e.g., electric field strength, flow rate, spray distance), and the ambient parameters (e.g., temperature, humidity) (Bhardwaj and Kundu, 2010; Pelipenko et al., 2015; Ye et al., 2019).

Core-shell nanofiber scaffold is a special type of nanofibers. Core-shell nanofiber can combine the excellent properties of two or more polymers. It uses the core-shell structure to encapsulate specific substances, such as drugs and growth factors, into nanofibers (Kim et al., 2004; Bölgen et al., 2007; Nie and Wang, 2007). In the fiber material system, nanofibers have the characteristics of high porosity, high specific surface area, and high surface activity. This structure is suitable for processes in which the fiber needs to fully contact and react with the surrounding environment, such as drug release. Mixing the drug directly into the nanofibers often results in burst release of the drug, while the core-shell nanofiber can encapsulate drugs in the core layer and prolong their release (Mohiti-Asli et al., 2017; da Silva et al., 2019). Some drugs or active substances should not be exposed to organic solvents. The core structural material can enclose the drugs or active substances inside to prevent them from being exposed to organic solvents. Because the drug or active substance is dissolved in the core solution, the surface modification of the fiber will not affect the activity of the internal drug. The main manufacturing methods of core-shell nanofibers reported in the literature are coaxial electrospinning and emulsion electrospinning.

#### 2.1.1 Coaxial electrospinning

The difference between coaxial electrospinning and ordinary electrospinning lies in the design of the spinneret. Ordinary electrospinning uses a single-layer capillary while coaxial electrospinning uses a coaxial nozzle (Zupančič et al., 2016). A coaxial nozzle is formed by nesting two or more coaxial capillaries with each other, and a certain gap is left between the inner and outer capillaries to ensure the smooth flow of the shell liquid. In coaxial electrospinning, the solution of the core and shell material is divided into two different syringes, and the spinning system is composed of two coaxial but different inner diameter capillary tubes. Under the action of a high-voltage electric field, the outer shell liquid flows out (Huang et al., 2006). Coaxial electrospinning relies on complex electrospinning equipment such as coaxial nozzles.

#### 2.1.2 Emulsion electrospinning

Emulsion electrospinning is a technical method capable of preparing nanofibers with core-shell structure in one step (Bazilevsky et al., 2007; Moydeen et al., 2018). In general, emulsion electrospinning solution mainly uses a water-in-oil or oil-in-water two-phase dispersion system to form an external phase by continuous polymer organic solution and an internal phase with drug aqueous solution in the emulsion. Amphiphilic emulsifiers are used to stabilize the interaction between the external phase and the internal phase to form an emulsion (Qi et al., 2006). The drug-loaded nanofibers prepared by this method can effectively solve the problem of burst drug release and can slow and control the release of the loaded substance (Liu et al., 2018). Compared with coaxial electrospinning, the advantage of emulsion electrospinning is that the device is simple without the requirement of coaxial nozzles. However, it has higher requirements for spinning solution, and the instability of the emulsion limits its wide application.

### 2.2 3D printing

**FIGURE 3:**
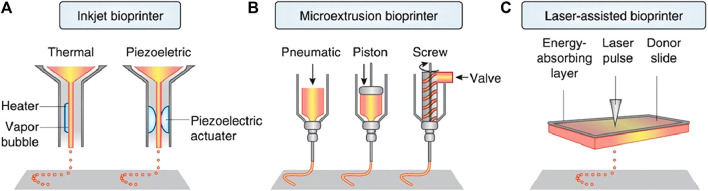
Three main 3D bioprinting technologies. **(A)** Inkjet printing; **(B)** Extrusion or robotic dispensing bioprinters; **(C)** Laser-assisted bioprinting (Bartolo et al., 2022).

3D printing technology, a type of rapid prototyping technology, is a digital model file based on the use of adhesive materials through layer-by-layer printing to construct an object. As a new technology, 3D printing is developing rapidly, as there are many established manufacturing techniques and a large number of experimental techniques (Li et al., 2020). The most popular 3D bioprinting technologies include laser-assisted bioprinting, inkjet printing, and extrusion and robotic dispensing bioprinters (Figure 3). In recent years, 3D printing technology has been applied to tissue engineering with the aim of constructing biomaterials that simulate the microstructure of tissues and organs. The 3D bio-printing process comprises three steps. (1) Data collection and software modeling. This mainly includes 3D image collection and digital 3D design; (2) choice of printing ink (materials and cells); and (3) improvement of the biomimetic structure, mechanical properties, and biological activity of the scaffolds (Zhu et al., 2016).

At present, many materials such as synthetic polymer materials (polyethylene glycol (PEG), polylactic acid (PLA), polycaprolactone (PCL), polyetheretherketone (PEEK)) and natural polymer materials (collagen, gelatin, hyaluronic acid) have been used in 3D printing. However, the accuracy of 3D printing is limited. Generally, it is used to print micron-sized materials; therefore, it is often necessary to combine it with other technologies to obtain tissue engineering scaffolds with higher accuracy.

### 2.3 Microfluidic technology

**FIGURE 4:**
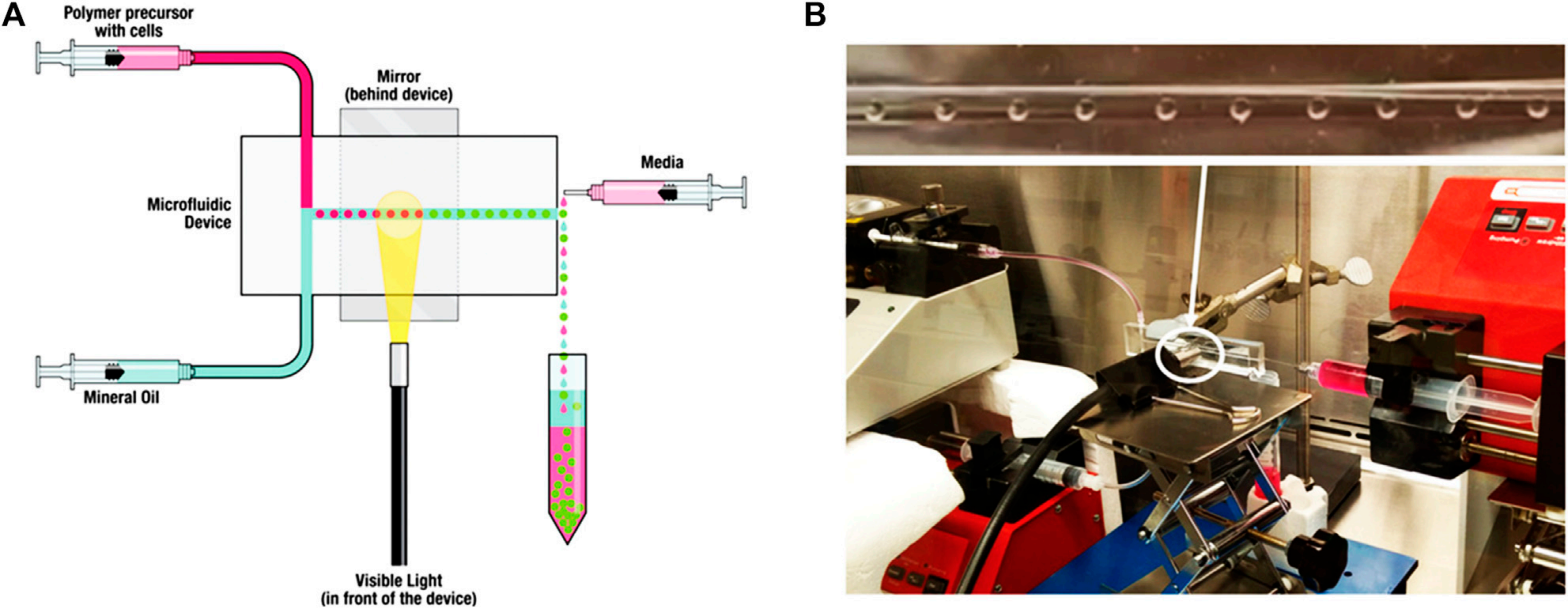
Microfluidic encapsulation platform using a novel custom design and device molding technique enables production of uniform hydrogel microspheres with a wide range of diameters. **(A)** Schematic of the microfluidic encapsulation platform. **(B)** Setup of the microfluidic encapsulation platform in a biosafety cabinet (Seeto et al., 2019).

In recent years, the technological advances in materials science have contributed to the production of various micro/nano-materials such as microspheres for biomedical applications (Tien and Dance, 2021). Interestingly, the characteristics of microfluidic technology are helpful for preparing these micro-nano materials with precise control of reaction parameters in a short time and minimal reagent consumption. Generally, the synthesis of micro-nano materials in microfluidic platform can be achieved by mixing single or multiple miscible solvents (single-phase microfluidic) or mixing multiple immiscible solvents (multiphase microfluidic). The microfluidic reaction system has become a high-throughput and controlled tool, which can be used for materials with diverse biological and chemical applications. Due to the typical laminar flow environment of microfluidic channels, the reaction can be carried out in a highly controlled and reproducible manner (Domachuk et al., 2010). In addition, the size and composition of the product can be flexibly designed by adjusting the flow rate, characteristic geometry and the properties of the input reagent. Different from conventional methods, microfluidic technology allows the continuous production of new materials in a single workflow. Most importantly, many reactions in the microfluidic platform can be carried out under mild conditions, which is very beneficial to many bioactive reaction systems. Therefore, researchers use microfluidics to prepare a large number of materials for biological applications, including microspheres for biomedical applications (Figure 4), microcapsules for medical diagnosis applications, and micron (or nano) fibers for tissue engineering.

## 3 Types of micro/nano-materials

### 3.1 Fibers

**FIGURE 5:**
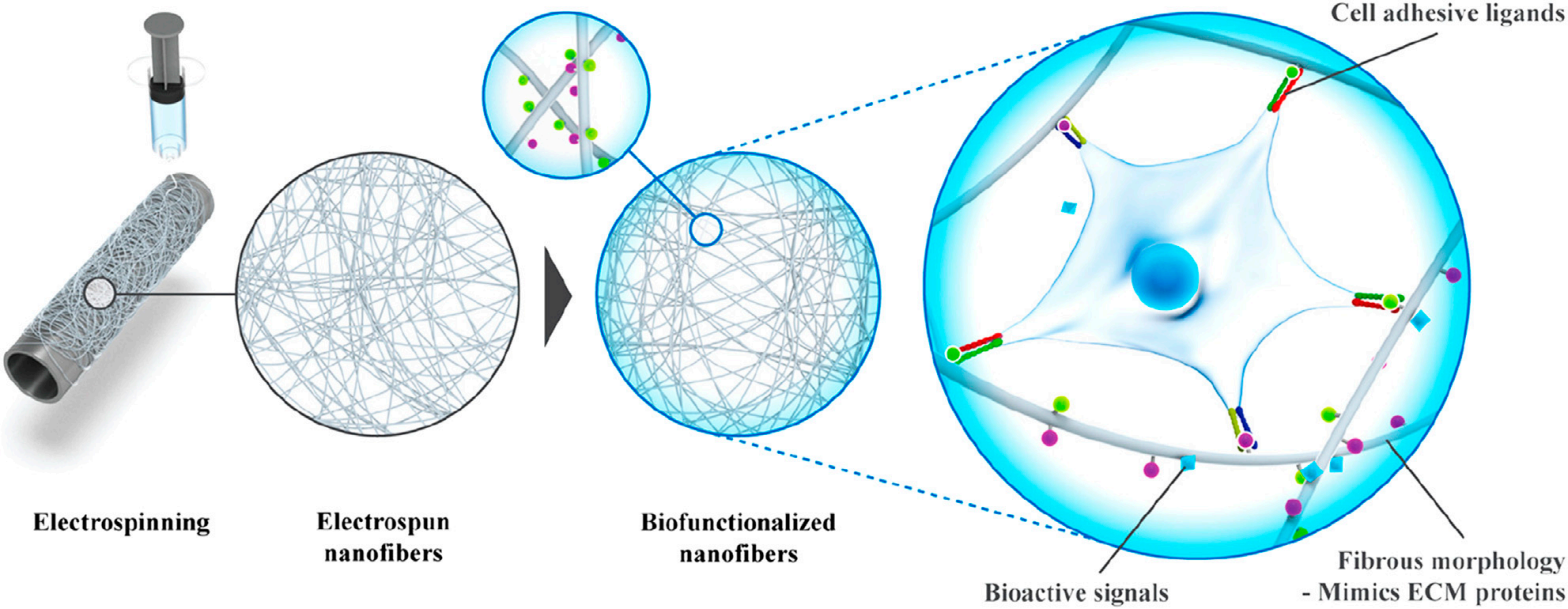
Fabrication of bioactive electrospun fibers mimicking the native ECM (Taskin et al., 2021).

Electrospinning is a feasible technology to produce nanofibers, which can be prepared from various types of biomaterials (Xue et al., 2019). The most commonly used materials are synthetic polymers, but natural polymers such as alginate are also used. In addition, synthetic materials offer many advantages over natural polymers, such as reliable sources, low immunogenicity and cost-effectiveness. Biodegradability and structural modifiability are additional advantages of synthetic polymers. The most commonly used polymers include PLA, polyglycolic acid (PGA), PCL, polyurethane (PU), poly (vinyl alcohol) (PVA) and poly- (ethylene oxide) (PEO). The microstructure of nanofibers is also very important for tissue repair and regeneration. Compared with random PCL nanofibers, aligned nanofibers have stronger AF repair effect (Gluais et al., 2019). Besides the arrangement of nanofibers, the stiffness and fiber size of nanofibers will also affect AF stem cell differentiation. According to different needs, specific stiffness and fiber size can be designed to guide cells to differentiate in a specific direction, regulate cell morphology and promote the expression of related ECM (Chu et al., 2019). To further improve the mechanical properties of nanofibers and enhance the interaction with cells, synthetic polymers are also often functionalized through the combination of two or more materials. Nanofibers can also be modified with bioactive cues such as ECM proteins, growth factors, peptides, and nucleic acids to control the cell attachment, migration, spreading, proliferation, and differentiation (Figure 5) (Taskin et al., 2021).

When hydrophobic materials such as PCL are delivered to the body, immunological rejection may occur, resulting in unspecific protein adsorption and an inflammatory response (Ratner and Bryant, 2004). The incorporation of hydrophilic bioinert materials such as PEG, can effectively shield hydrophobic fibers, thus making the biomaterial resistant in the biological environment (Dash and Konkimalla, 2012). In addition, the surface of poly (L-lactic acid) (PLLA) scaffolds treated with plasma is more hydrophilic (Jin Seo et al., 2013). It was shown that PCL-α-CD fibers obtained from PCL co-electrospinning with cyclodextrins (CDs) promoted osteogenic differentiation of human adipose-derived stem cells compared with pristine PCL fibers (Zhan et al., 2012). The plasma-treated polyester fiber further improves the adhesion and proliferation of human mesenchymal stem cells (hMSCs), fibroblasts, osteoblasts and Schwann cells (Jia et al., 2008; Martins et al., 2009; Nandakumar et al., 2013). Moreover, studies have confirmed that polydopamine-coated nanofibers (e.g., PCL, PLLA and PLGA) support the deposition of serum proteins, accelerate cell attachment and are widely used for bone, cardiac, skin, nerve and vascular tissue engineering (Chen et al., 2007; Taskin et al., 2016). Furthermore, a variety of active proteins can be modified to nanofibers to enhance the biological activity of fibers, such as vascular endothelial growth factor (VEGF) and epidermal growth factor (EGF) (Choi et al., 2008; Li et al., 2008; Guex et al., 2014; Heo et al., 2015). For instance, VEGF was immobilized on a nanofibrous scaffold and effectively stimulated endothelial cell proliferation (Guex et al., 2014). Shin et al. demonstrated that gelatin and EGF immobilized together on acrylic acid-grafted poly (lactide-co-ε-caprolactone) (PLCL) significantly enhanced the ability of hMSCs to differentiate into keratinocytes (Shin et al., 2015). Stable and self-assembled coatings on the surface of nanofibers play an important role in the delivery system, enabling precisely temporal release of encapsulated molecules (Yoo et al., 2009; Shah et al., 2011). For instance, polylaminate coating on PCL nanofibers can be used for the quick release of connective tissue growth factor (CTGF) and to regulate the release of bone morphogenetic protein-2 (BMP-2), with the aim of mimicking the natural bone healing process (Cheng et al., 2019). Functionalization of nanofibers can also be achieved through click chemistry (Callahan et al., 2013). The introduction of RGD peptide and osteogenic growth protein (OGP) on PCL nanofibers promoted the differentiation of preosteoblast cells into osteoblasts (Kim et al., 2015).

In addition to electrospinning, microfibers can be prepared by microfluidics (Costantini et al., 2016; Zuo et al., 2016; Wang et al., 2019). Zuo et al. designed a dual coaxial capillary microfluidic device that mimics the unique structure of the osteon (Zuo et al., 2016). The middle and outer layers of the fabricated bilayer hollow microfibers are composed of human umbilical vein epithelial cells (HUVECs) and human osteoblast-like cells (MG63), respectively, imitating the vascular layer and bone tissue. Furthermore, the microfibers consist of an alginate-GelMA composite hydrogel in the middle and outer layers, and hyaluronic acid in the inner layer. The incorporation of GelMA reduces the concentration of alginate and improves the biocompatibility of the composite hydrogel, without altering the mechanical properties of the composite hydrogel. Topological hydrogel microfibers have been successfully used to mimic muscle-like bundles (Costantini et al., 2017; Miri et al., 2018; Bansai et al., 2019). Bansai et al. demonstrated that C2C12 muscle precursor cells reorganized their cytoskeleton and formed longitudinal myofibrillar-like structure when wrapped in the collagen-rich core of hydrogel core-shell microfibers (Bansai et al., 2019). Moreover, the alginate shell enhances the mechanical stability of the microfibers and promotes C2C12 cell elongation and myogenesis.

The structure, physical and chemical properties and biological effects of nanofiber scaffolds are of great significance to the development of tissue engineering scaffolds. Although some achievements have been made in the preparation and research of nanofiber materials, how to improve the preparation process, shorten the preparation period of materials, reduce the processing cost of materials, increase the porosity of scaffold materials and control the size and distribution of pore size needs further study. In addition, ECM contains both micron pores and nano-space. There is a synergistic effect between them, which provides the necessary space for cell planting, growth and ECM formation, and provides channels for oxygen and nutrition transmission, information transmission, gene expression and metabolite excretion. Therefore, the ideal scaffold not only needs to be composed of continuous nanofibers with sufficient mechanical properties, but also needs to contain both micro and nano spaces. But up to now, we still lack sufficient understanding of the optimal fiber diameter and its corresponding pore size and distribution, the ideal ratio of nano-space to micro-space, and the interaction between nano-space and body tissue. It is believed that in the future, with the development of nanotechnology and its continuous penetration in the field of tissue engineering, there will be a breakthrough in the design, preparation and performance regulation of nanofiber scaffolds.

### 3.2 Microspheres

Microspheres have become advanced functional materials used in a wide range of biomedical applications, such as researchers prepared microcapsules and microcarriers as a vehicle for drug delivery, summarized the recent progress of engineering particles and their emerging applications in biomedical delivery and diagnosis, and reviewed a comprehensive and in-depth insight into droplet microfluidics, covering fundamental research from microfluidic chip fabrication and droplet generation to the applications of droplets in bio (chemical) analysis and materials generation (Hernandez et al., 2010; Choi et al., 2017; Shang et al., 2017). It is suggested that the distribution of microsphere-sized particles is thought to be an important factor affecting the choice of drug delivery route and the rate of drug release. The properties of microspheres are closely related to their size, structure, and composition; therefore, it is essential to manufacture microparticles in a controlled manner to improve the reliability of their application (Tran et al., 2011; Duncanson et al., 2012; Thiele, 2017). Currently, most of the custom microspheres are manufactured by microfluidics and electrohydrodynamic co-jetting (Yeh et al., 2006; Dendukuri and Doyle, 2009; Seo et al., 2015). Moreover, microspheres can be prepared from a host of materials, including nature polymer (collagen, gelatin, hyaluronic acid) and synthetic polymer (PEG, PLGA, polyglycerol, poly (acrylic acid) and poly (acrylamide)) (Velasco et al., 2012; Rossow et al., 2017).

Microspheres produced by droplet microfluidics can be used as excellent drug delivery microcarriers. To ensure the effectiveness of drug encapsulation, it is necessary to select the material with great compatibility with the drug. For instance, chitosan-based microspheres enable ampicillin or bovine serum albumin (BSA) encapsulation (Yang et al., 2007; Xu et al., 2012). Microspheres with core-shell structure allows the drug to be encapsulated in the core, while the shell acts as a diffusion barrier and controls the release curve (Lee et al., 2017). In addition, microspheres with pH-responsive properties show great potential for targeted drug delivery. Core-shell alginate microspheres tolerate acidic environments and degrade in alkaline environments while releasing encapsulated substance for intestine-targeted drug delivery (Huang et al., 2014). Hydrogel microspheres are exciting platforms for cell culture, because they act as 3D matrices that mimic ECM (Shum et al., 2008; Velasco et al., 2012; Zheng et al., 2023). In addition, hydrogel microspheres with porous structures and the high surface-to-volume ratios are conducive to the exchange of oxygen and nutrients between cells and the external environment, while maintaining cell activity. These advantages allow for the potential use of cell-loaded hydrogel microspheres in the field of cell life research (Huang et al., 2017; Rossow et al., 2017), drug delivery (Shembekar et al., 2016; Agarwal et al., 2017; Zhao et al., 2021), and tissue engineering (Chung et al., 2012; Jiang et al., 2016). For instance, a 3D liver model made of heterotypic cells and core-shell microspheres was assembled (Chen et al., 2016). The layered assembly of hepatocytes in the core and fibroblasts in the outer shell leads to the formation of a spheroid of allotypic cells. The alginate shell allows the spheroid to be cultured for long periods, and the hepatocytes and fibroblasts to be spatially separated and co-cultured, which facilitates the expression of liver-specific functions. Furthermore, microspheres containing sensing components prepared by microfluidics have been used in sensing applications (Le Goff et al., 2015). Fluorescent polyacrylamide hydrogel microspheres are sensors with high sensitivity, biostability, durability, and injectability for continuous monitoring of *in vivo* blood glucose levels (Shibata et al., 2010).

However, there are still many problems in the preparation of microspheres, such as (1) the size of microspheres is difficult to be unified and the size distribution is uneven; (2) microspheres are easy to aggregate; (3) the preparation cost of microspheres is high. Therefore, it can be predicted that the future research trends of microspheres may be: (1) exploring how to unify the size of microspheres to improve the preparation efficiency; (2) choosing suitable methods to disperse the microspheres to prevent them from aggregation; (3) reducing the preparation cost of microspheres; (4) Further broadening the clinical application of microspheres.

### 3.3 Nanoparticles

Nanoparticles with high specific surface area and excellent encapsulation capabilities have a wide range of applications, including drug delivery, sensors, bioimaging, and catalytic and diagnostic systems (Chen et al., 2016; Li et al., 2017). Nanoparticle-based drug delivery systems offer significant advantages over traditional drugs, such as increasing drug solubility and stability, achieving controlled drug release, and overcoming biological barriers (Cui et al., 2016). The major classes of materials used to construct nanoparticles include lipid, polymers and inorganic materials (Ju et al., 2022).

**FIGURE 6:**
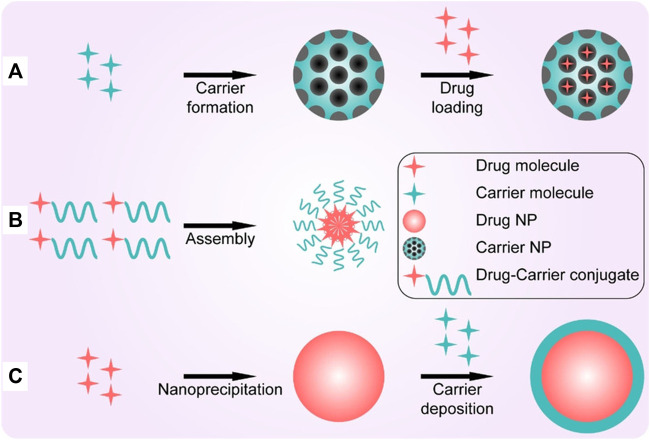
Three representative strategies for making high drug-loading nanoparticles. **(A)** Post-loading; **(B)** co-loading; **(C)** pre-loading (Liu et al., 2020).

Lipid nanoparticles (LNanoparticles), with a liposome-like structure, are widely used for the delivery of drugs (Mitchell et al., 2021; Eygeris et al., 2022). Nanoparticles can achieve drug loading in different ways including post-loading, co-loading, and pre-loading (Figure 6) (Liu et al., 2020). The efficacy of LNanoparticles for drug delivery, together with their simple synthesis, small size and serum stability, allow LNanoparticles to play an important role in personalized gene therapy, and in the penetration of the blood-brain barrier (Berraondo et al., 2019; Cheng et al., 2020; Hou et al., 2021).

Polymeric nanoparticles can be formed from natural or synthetic materials and monomeric or pre-polymeric polymers to effectively deliver a wide range of drugs, including hydrophobic and hydrophilic compounds, and carriers of varying molecular weights, allowing the precise control of the loading efficiency and release kinetics of these therapeutic agents by modulating properties such as composition, stability, reactivity, and surface charge (Caldorera-Moore et al., 2019; Knight et al., 2019; Zhang et al., 2020). PLGA has been approved by the FDA for its excellent biosafety, biodegradability and versatility, and is considered to be one of the most successful polymers in the biomedical field (Ulery et al., 2011; Martins et al., 2018; Niza et al., 2021). PLGA-based micro/nanoparticles have been used as carriers for small molecule and macromolecular drugs, with therapeutic benefits in diverse conditions such as diabetes or CNS related diseases (Bala et al., 2004; Rezvantalab et al., 2018; Essa et al., 2020; Ghitman et al., 2020). Furthermore, recent studies have proposed that PLGA itself can act as an active molecule. For instance, impaired lysosomal acidification occurs in neurodegenerative diseases, leading to lysosomal dysfunction (Udayar et al., 2022). This negative effect can be offset by the products of PLGA cleavage, such as lactic acid and glycolic acid (Bourdenx et al., 2016; Zeng et al., 2019). Bourdenx et al. demonstrated that restoration of lysosomal function significantly reduced substantia nigra dopaminergic neurodegeneration (Bourdenx et al., 2016). Moreover, Paul et al. verified that PLGA nanoparticles could inhibit Aβ aggregation and improved the cell survival rate of cortical neurons in Aβ1-42 treated mice (Paul et al., 2022).

With the increase in research on silk sericin, it was found that silk sericin has remarkable biological functions and can be used as a potential biomaterial (Liu et al., 2022). Numerous studies have indicated that silk sericin has low immunogenicity and does not cause significant immunoreaction (Chouhan and Mandal, 2020; Siavashani et al., 2020; Ode Boni et al., 2022). In addition, silk sericin with antioxidant properties has been shown to regulate glycolipid metabolism and promote the proliferation of various cell types while maintaining their functions (Kato et al., 1998; Ersel et al., 2016; Jena et al., 2018). Due to their good bioactivity and biocompatibility, sericin-based nanomaterials are currently being widely used in tissue engineering. Sericin-based nanoparticles act as nanocarriers for insoluble molecules, providing curative effects and reducing side effects (Kanoujia et al., 2016; Suktham et al., 2018). In addition to being a drug carrier, silk sericin serves as a biomineralization matrix (Wang et al., 2017; He et al., 2017; Chaisabai et al., 2018; Tian et al., 2020). For instance, nucleation of hydroxyapatite (HA) crystals can be mediated by using sericin as a template (Yang et al., 2014).

Inorganic materials can be designed in a wide range of sizes, structures, and geometries, and for various drug delivery and imaging applications (Mitchell et al., 2021). CaCO_3_ nanoparticles have the characteristics of biocompatibility and biodegradability, and controlled synthesis and easy functionalization (Trofimov et al., 2018). In addition, as a porous microcarrier, CaCO_3_ nanoparticles can be used for drug delivery. Animal and clinical studies have shown that nasal delivery of porous CaCO_3_ microcarrier can rapidly and effectively control blood glucose levels in diabetic patients (Haruta et al., 2003). No drug loss was observed when nifedipine, ibuprofen and other drugs with poor water solubility loaded onto CaCO_3_ nanoparticles (Preisig et al., 2014). In addition, Ca^2+^ produced by the decomposition of CaCO_3_ may serve as exogenous calcium sources to promote tissue repair, such as bone formation (Combes et al., 2006; Zhong et al., 2016).

Iron oxide is a commonly studied inorganic material and nanomedicines made from iron oxide nanoparticles are approved by the FDA (Bobo et al., 2016). Magnetic iron oxide nanoparticles consisting of magnetite (Fe_3_O_4_) or maghemite (Fe_2_O_3_) have superparamagnetic properties at specific sizes and have shown excellent promise as drug delivery carriers, contrast agents and thermotherapy agents (Arias et al., 2018). As common inorganic nanoparticles, silicon quantum dots are mainly used as unique nanoparticles for imaging applications *in vitro*, but they show prospect for *in vivo* diagnostics (Xu et al., 2019; Huang et al., 2020). Due to the different oxidation states of cerium (Ce^3+^/Ce^4+^), cerium oxide (CeO_2_) nanoparticles have excellent resistance to oxidation, which has been reported in the treatment of diseases, such as spinal cord injury, inflammation, sepsis and Alzheimer disease (AD) (Selvaraj et al., 2015; Kwon et al., 2016; Kim et al., 2017). For instance, in a mouse model of AD, small and positively charged CeO_2_ nanoparticles conjugated with triphenylphosphonium attenuated neuronal death, alleviated mitochondrial damage, and reduced reactive gliosis by scavenging reactive oxygen species (ROS) (Kwon et al., 2016).

### 3.4 3D printing scaffold

Modulation of macro, micro and nano structures can be achieved by bioprinting strategies (Tarassoli et al., 2018; Chakraborty et al., 2022). 3D bioprinting enables the rapid and precise spatial patterning of cells, growth factors and biomaterials to create complex 3D tissue structures (Matai et al., 2020; Kim et al., 2021). The printed structures should have excellent biocompatibility, great mechanical and biomimetic properties. To achieve this, a variety of bioinks are used for bioprinting, such as fibrinogen, agarose, alginate, collagen, gelatin, hyaluronic acid, pluronic or poly (ethylene glycol) (Shao and Vollrath, 2002; Muller et al., 2015; Wang et al., 2015; Axpe and Oyen, 2016; Mandrycky et al., 2016).

3D porous polymer scaffolds have been used to generate substitutes for skin tissue. A 3D bioprinted skin tissue model was developed using silk fibroin-gelatin (SF-G) bioink and human dermal fibroblasts, keratinocytes and melanocytes (Admane et al., 2019). The 3D bioprinted structure mimics the morphology of the dermal-epidermal junction and replicates the mechanical properties and biochemical characteristics of human skin, ultimately facilitating full-thickness wound healing (Xiong et al., 2017; Admane et al., 2019). In addition, 3D bioprinted skin tissue equivalents serve as *in vitro* models to test the biosafety of pharmaceuticals and cosmetics, contributing to the study of cell signaling and physiological response mechanisms (Chakraborty et al., 2022).

Multiple causes, such as trauma, can lead to bone defects. However, no consistent results have been achieved in terms of bone replacement in current bone tissue engineering strategies. Although it is challenging to simulate the complex anatomical structure and functional dynamics of native bone, 3D bioprinting can be a potential method for bone tissue replacement (Kacarevic et al., 2018). For instance, 3D printed scaffolds through combining SF, gelatin, hyaluronic acid and tricalcium phosphate (TCP) can support osteogenic differentiation of human adipose-derived mesenchymal stem cells (Du et al., 2019). It has also been shown that bioprinted BMSC-loaded GelMA/SFMA scaffolds can promote bone repair through vascularized osteogenesis (Yang et al., 2022). The microstructure of intervertebral disc is complex, which can be simulated by 3D printing technology. 3D printing of flexible polylactic acid (FPLA) can be used to fabricate a viscoelastic scaffold with tunable biomimetic mechanics for whole spine motion segment applications (Marshall et al., 2021).

Although 3D printing technology has many advantages that other materials cannot achieve, the types of printing materials are still limited, which is the main bottleneck that hinders the development of 3D printing technology. At present, the main way of 3D printing is to print materials in the form of “ink” and quickly solidify them. Therefore, while “ink” has good biocompatibility and biodegradability of general biomaterials, it should also be considered that after printing, it still needs to maintain its original biological activity and mechanical strength, which greatly limits the application scope of 3D printing. The existing 3D printing equipment also has low printing precision and cannot meet the bionic requirements of tissues and organs.

### 3.5 Others

#### 3.5.1 Micro-/nano-structured smart hydrogels

Smart hydrogels, a cross-linked polymer, can respond to small changes in environmental stimuli by significantly altering their volumes and other physico-chemical properties. The uniqueness of these hydrogels lies in their non-linear feedback. They respond to external stimuli primarily through reversible, intensity-scalable, repeatable and predictable phase volume shifts and are able to return to their original state after the stimulus has been removed. These transformations include changes in physical state and hydrophilicity, etc (Bordbar-Khiabani and Gasik, 2022). In addition, the flexibility of different response behaviors in response to different stimuli is important for broadening the application of smart hydrogels in various conditions. It is worth noting that micro-/nano-structured smart hydrogels show higher elasticity and respond faster and stronger to external stimuli than smart hydrogels without micro-/nano-structures (Wang et al., 2021).

Poly (N-isopropyl acrylamide) (PNIPAM) hydrogels are commonly used thermal responsive hydrogels with reversible swelling/contraction changes when the temperature is varied within the volume phase transition temperature (VPTT) range. In general, PNIPAM hydrogels formed by chemical crosslinking of N, N′-methylenebisacrylamide (BIS) and monomer N-isopropylacrylamide (NIPAM) have a slow response and poor elasticity. However, when PNIPAM nanogels with unsaturated C=C bonds are used to cross-link PNIPAM chains, micro/nanostructured PNIPAM hydrogels with remarkable elastic and responsive properties can be obtained (Xia et al., 2013). The transition from the fully swollen to the fully collapsed state of the nanogel cross-linked hydrogel takes only 6 min, much faster than the normal PNIPAM hydrogels. Moreover, the swelling ratio of nanogel cross-linked hydrogel is 10 times that of the normal PNIPAM hydrogel. In addition to responsive volume changes, smart hydrogels can achieve various stimuli responses (Isapour and Lattuada, 2018; Wen et al., 2019; Jia et al., 2021). For instance, it is often possible to obtain smart hydrogels with responsive color changes by designing colloidal nanocrystal structures in their polymer networks (Jia et al., 2021). BIS and graphene oxide nanosheets with photothermal properties can be used as double crosslinking agents to produce PNIPAM hydrogels with micron-scale network. This double cross-linked hydrogel allows ultra-high stretchability and rapid volume phase transition in response to NIR light (Wang et al., 2022).

#### 3.5.2 Micro-/nano-scale liquid metal

**FIGURE 7:**
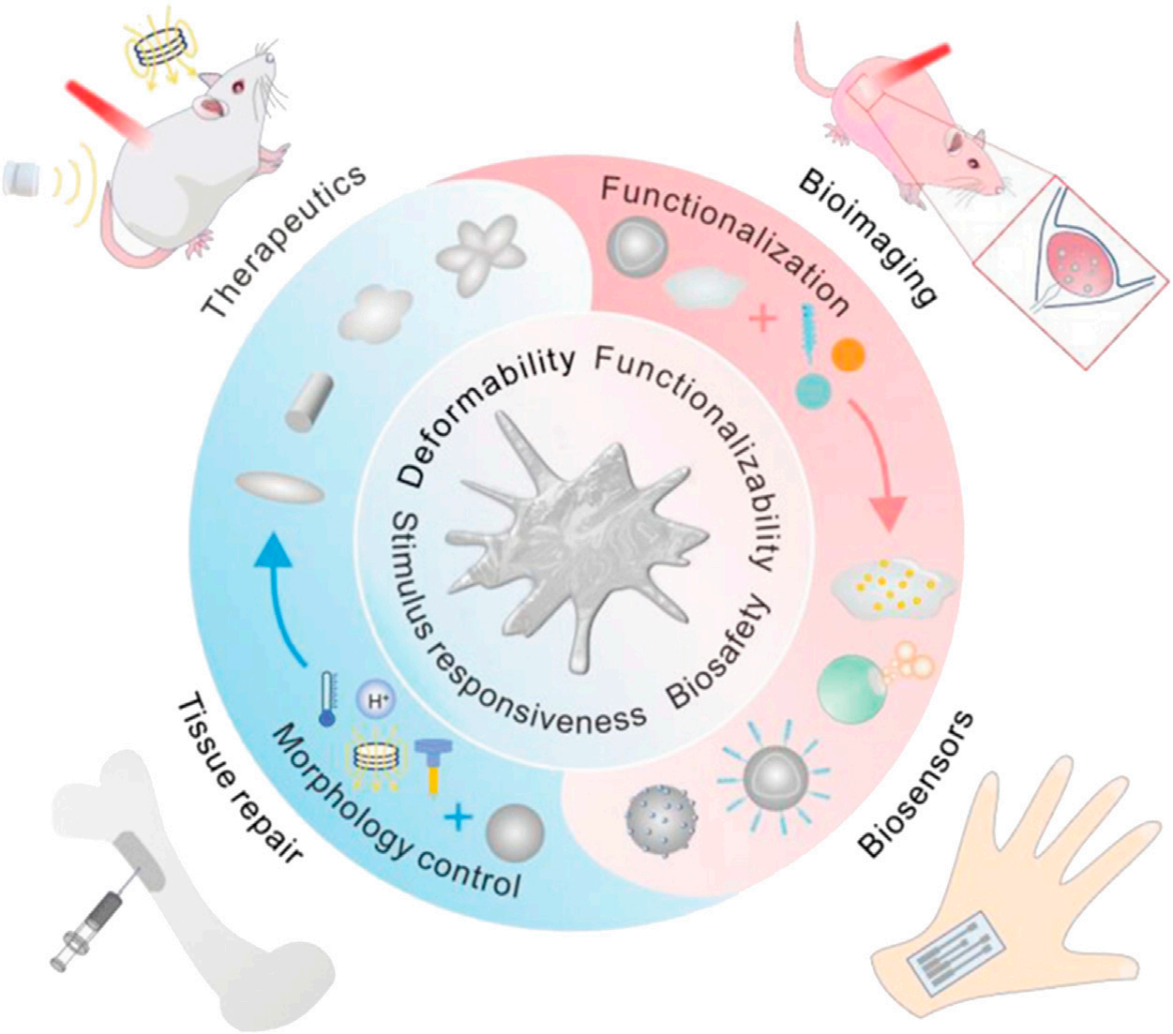
Schematic illustration of advantages and design of LM and their applications in biomedicine (Wang et al., 2022).

As a new biological material, liquid metal (LM) has shown promising prospects in the biomedical field, such as tissue therapeutics, repair, bioimaging, and biosensors (Yan et al., 2018; Zeng and Fu, 2018; Miyako, 2021). LM with preeminent deformability can be molded into various shapes with different properties to cope with diverse application scenarios (Wang et al., 2018; Sun et al., 2019). In addition, the morphology of LM changes significantly in response to external stimuli such as pH, light, temperature, and magnetic fields. Additionally, the morphological changes induced by stimuli can help to expand the application of LM in drug delivery, thermotherapy and antimicrobial therapy (Wang et al., 2022) (Figure 7). For instance, LM nanoparticles generally have an oxide layer on their surface, which is due to self-limiting oxidation. The oxide layer can react with protons and dissolve in an acidic environment. Therefore, drug release from LM can be facilitated in an acidic microenvironment due to the removal of the oxide layer (Lu et al., 2015). Furthermore, under NIR irradiation, the shape of polydopamine (PDA)-coated LM nanoparticle changes from spheres to ellipsoids (Gan et al., 2019). Based on this transformation, the prepared LM nanorobots exhibit excellent photothermal properties and can be used for photothermal antibacterial therapy (Xu et al., 2021). LM with good biocompatibility has emerged as a potential material for tissue repair, including bone defect repair and nerve connectors (Yi et al., 2014; Liu et al., 2016; He et al., 2021). LM used in biomedicine generally include gallium (Ga) and its alloys, which can achieve solid-liquid transition at relatively low temperatures to satisfy the needs of different application environments via the component modulation. Kinds of Ga-based biomaterials such as Ga droplets and Ga-based hybrids show low toxicity in aqueous environments and mice bodies. At the same time, Ga-based LM possess good degradability in acidic biological environments, thus helping to reduce their potential systemic toxicity. In particular, Ga ions also exhibit antibacterial abilities and can be used to treat bacterial infections. In addition, an actuator using LM based on Ga can circumvent the problems of conventional actuators such as high drive potential, low strain and their time responsiveness. Thus, Ga and its alloys have great potential for tissue engineering applications (Wang et al., 2022).

## 4 Applications of micro/nano-structured materials for tissue regeneration

Micro/nanomaterials can simulate the microstructure of tissues and can also be used as carriers of drugs and growth factors. Research on micro/nanomaterials in tissue repair and regeneration is gaining increasing attention. Micro/nanomaterials play a very important role in the treatment of bone defects, cartilage injuries, cardiovascular diseases, skin injuries, intervertebral disc degeneration, and other diseases.

### 4.1 Bone

Bone tissue defect is caused by trauma, tumor and other diseases, and has become a common clinical disease. Currently, the use of orthopedic implants is an available option for treating these diseases. The use of bone grafts is on the rise, with more than 2.2 million cases of bone grafting worldwide each year (Wang and Yeung, 2017). In recent years, the development of nanotechnology, biomaterial science and tissue engineering has provided a broad prospect for the preparation of bone graft materials (Liu et al., 2022). Natural bone tissue has a unique structure, mainly composed of collagen, nanofibrils and nano-hydroxyapatite (Laubach et al., 2022). The use of micro-nanomaterials can play a key role in accelerating cell responses and guiding tissue regeneration. Therefore, it is necessary to construct bone tissue engineering scaffolds with consideration of aspects such as surface morphology, mechanical strength, and regulation of bioactive molecules. As a 3D scaffold for cells, bone tissue engineering scaffolds provide cells with the unique microstructure and microenvironment of bone tissue to maintain the survival and differentiation of cells (Park et al., 2016).

**FIGURE 8:**
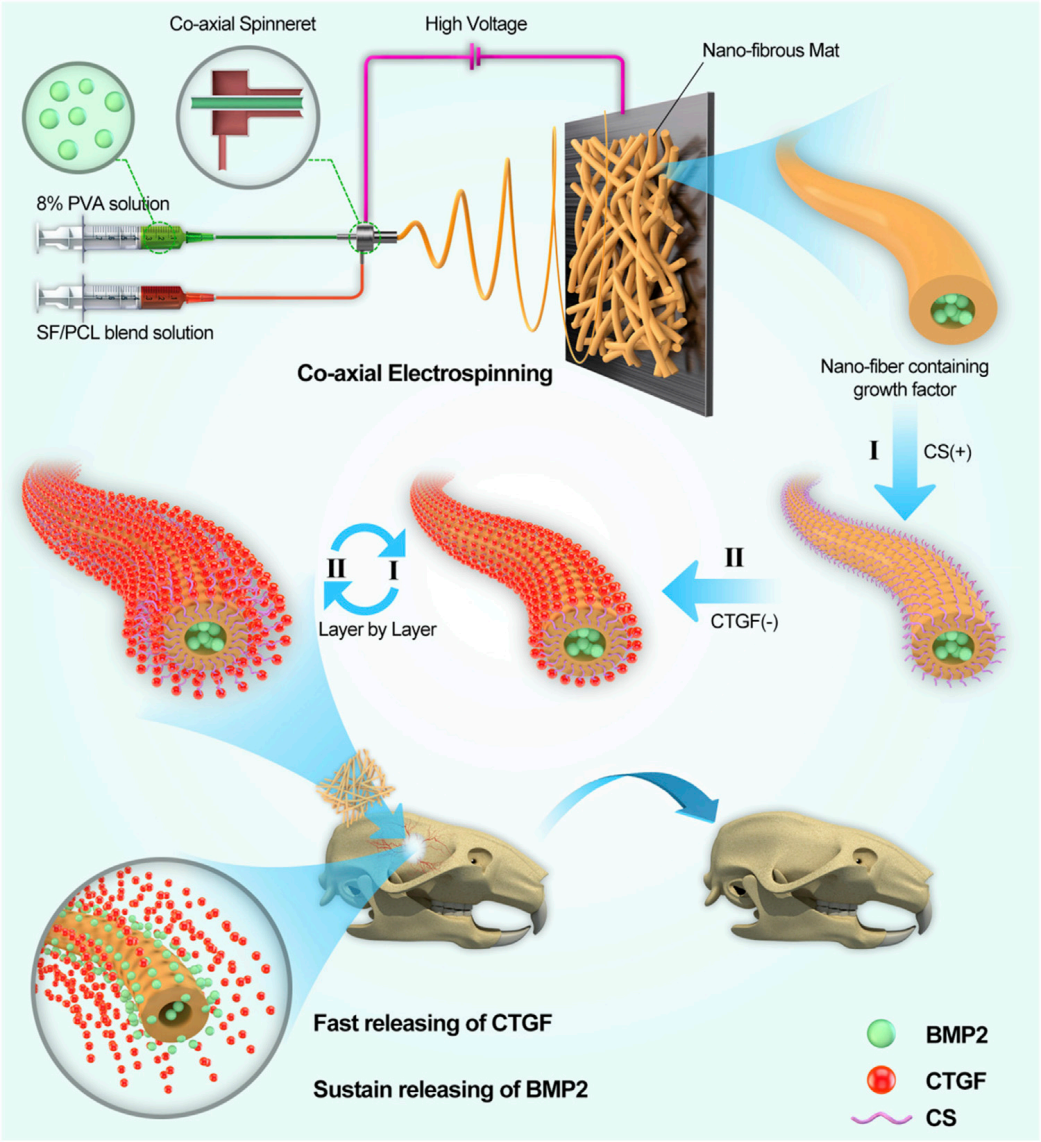
Schematic illustration of the (SF/PCL)_1:5_/PVA-LBL20 coaxial fibers loaded with BMP2 and CTGF for bone tissue engineering (Cheng et al., 2019).

Nanofiber scaffold is a commonly used scaffold for bone tissue engineering (Shalumon et al., 2015). Biomimetic composite scaffold of HA/gelatin-chitosan core-shell nanofibers has been used for bone regeneration, as it can mimic both the specific structure and the chemical composition of natural bone. MG-63 was seeded on the nanofibers and it was proved that the nanofibers could enhance osteoblast cell proliferation (Chen et al., 2019). Incorporation of dual factors, HA, and laminin within the shell and core of nanofibers respectively via emulsion electrospinning is another method of fabricating core-shell nanofibers. Nanofibers play important roles in osteoblast proliferation and maturation (Tian et al., 2013). Electrospun core-shell nanofibers loaded with metronidazole (MNA) and nano-hydroxyapatites (nHA) have both anti-infection and osteogenesis capabilities (Wang et al., 2019). Another type of core-shell nanofiber can incorporate an osteogenic inductive peptide H1, derived from the cysteine knot (CT) domain of CTGF, in the core of SF and co-deliver HA from the shell of poly (L-lactic acid-co-ε-caprolactone) (PLCL). It can promote proliferation and osteoblastic differentiation of human induced pluripotent stem cell-derived mesenchymal stem cells (hiPS-MSCs). *In vivo* experiments further verified that SF-H1/PLCL-HA core-shell nanofibers can promote the repair of bone defects (Xu et al., 2019). Platelet-rich plasma (PRP) can be incorporated into SF/PCL/PVA nanofibers by coaxial electrospinning to avoid the rapid degeneration of the growth factors, and this core-shell nanofiber can release multiple growth factors to promote bone regeneration (Cheng et al., 2018). BMP-2 is a growth factor commonly used in bone tissue engineering to promote bone regeneration. The core-shell nanofiber scaffolds can work as a sustained delivery vehicle for BMP-2 protein. The controlled release of BMP-2 can effectively promote the regeneration of bone tissue (da Silva et al., 2019). BMP-2 and other drugs or growth factors are often co-encapsulated in the core-shell nanofiber scaffold to promote bone regeneration. BMP-2 and dexamethasone (DEX) were successfully incorporated into PLLACL/collagen nanofibers by means of coaxial electrospinning. The controlled release of the two growth factors from PLLACL/collagen nanofibrous mats can induce hMSC to differentiate into osteoblasts for bone tissue engineering (Su et al., 2012). Zein/PLLA nanofibers can also be fabricated to load BMP-2 and DEX. It can achieve the burst release of DEX and sustained release of BMP-2. It can enhance the osteogenic differentiation of MSCs and has great potential in bone tissue engineering applications (Li et al., 2018). Core-shell SF/PCL/PVA nanofibrous mats using coaxial electrospinning and layer-by-layer (LBL) techniques, where BMP-2 was incorporated into the core of the nanofibers and CTGF was attached onto the surface were also fabricated. According to the physiological needs of bone regeneration, this core-shell nanofiber scaffold induced sustained release of BMP-2 and sudden release of CTGF. *In vivo* experiments found that this scaffold has the largest bone repair area, and the new bone formed has the same tissue structure as normal bone. Therefore, it can provide a promising strategy to facilitate bone healing (Figure 8) (Cheng et al., 2019). The mechanical properties of bone repair materials are required. Studies have shown that nHA and Fe_3_O_4_ nanoparticles can improve the mechanical properties of materials and further promote the repair and regeneration of bone tissue (Zhao et al., 2019).

3D printing technology is considered to be an advantageous technology, which can also be used to construct simulated bone tissue microstructure. In addition, scaffolds produced through 3D printing can provide excellent delivery vehicles for topical, continuous delivery of drugs and/or biomolecules. PLA/PCL/HA composite bone scaffold prepared by 3D printing can effectively simulate the microstructure of bone and has good biocompatibility (Hassanajili et al., 2019). Hierarchical porous and recombinant human bone morphogenetic protein-2 (rhBMP-2)-loaded calcium phosphate (Ca-P) nanoparticle/poly (L-lactic acid) nanocomposite scaffolds can also be fabricated by 3D printing, in which sustained releases of Ca^2+^ ions and rhBMP-2 can be achieved (Wang et al., 2017).

Microspheres and nanoparticles are also widely used in bone tissue engineering because of their good injectability and drug-controlled release ability and etc. nHA microspheres prepared via a hydrothermal transformation method significantly improved the ability of the microspheres to adsorb the bioactive protein (BMP-2) and realize bone regeneration (Zhou et al., 2018). rhBMP-2 can be grafted on the surface of mesoporous bioglass nanoparticles (MBGNs) with an amide bond. It can realize the rhBMP-2 release in a controllable program during the early bone regeneration period and then sustained release of calcium and silicon ions to keep promoting osteogenesis in a long term (Xin et al., 2020).

LM have good biocompatibility, deformability, good electrical and thermal conductivity and have become a potential material in the field of tissue repair. Severe bone defects caused by some diseases, such as bone tumors and external trauma, often require bone repair materials to replace the missing bone. Bismuth (Bi) alloys can be introduced as a bone defect repair material (bone cement) to fill the defect locations. Bi alloys have a low melting point and can be solidified at low temperatures to avoid damage to normal tissue. At the same time, the Bi alloys with its strong magneto-thermal effect can be utilized to alleviate mechanical and thermal pain sensitivity, so pain is relieved. This bone cement has a high affinity to bone and does not move significantly after implantation. Furthermore, the Bi alloys exhibited excellent imaging ability (Wang et al., 2022). In summary, LM have potential application in the field of tissue repair due to their unique advantages.

### 4.2 Cartilage

**FIGURE 9:**
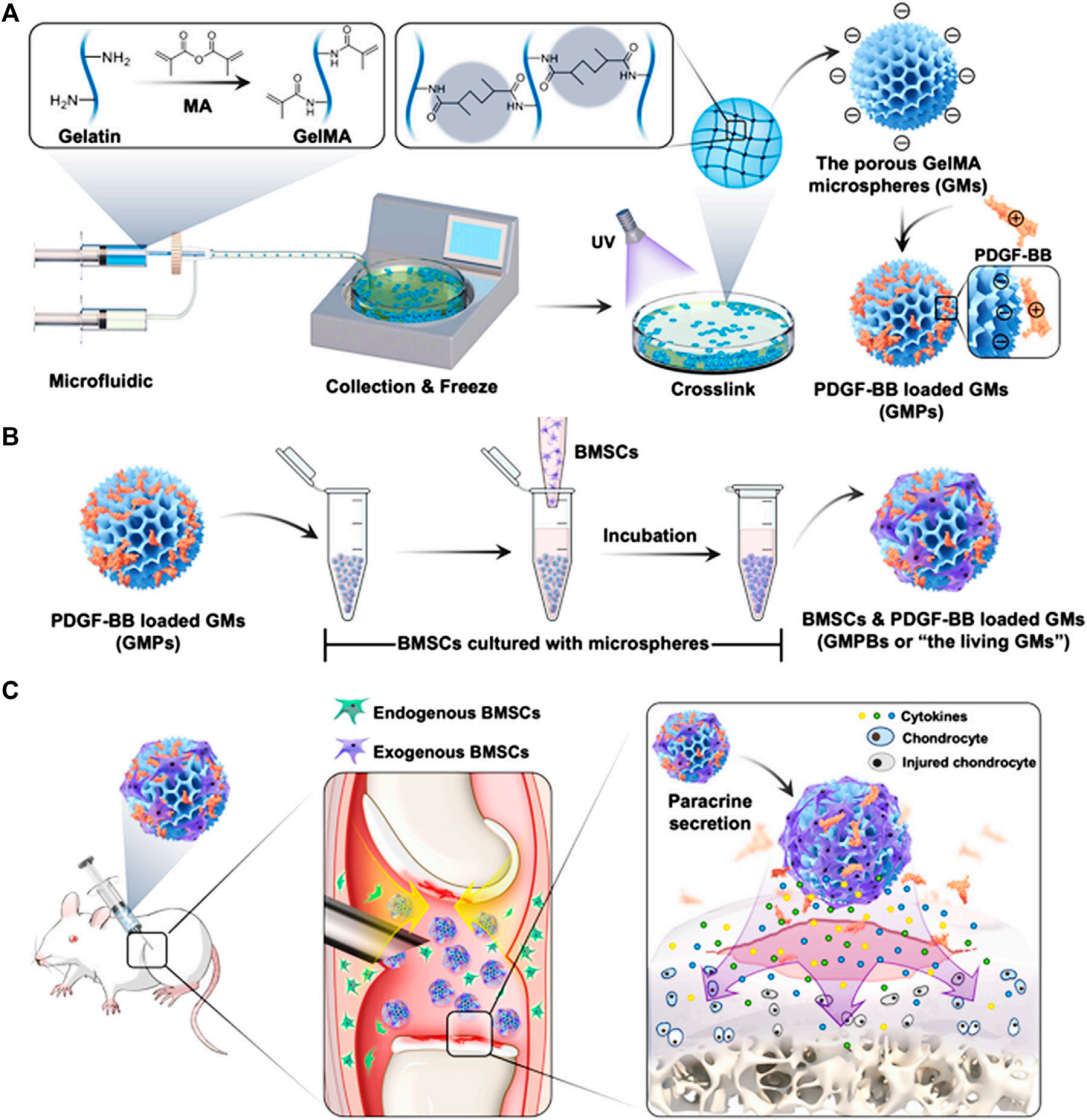
Schematic illustrations of the living GMs for cartilage regeneration. **(A)** The fabrication of GMs using microfluidic technology, and incorporation of PDGF-BB via the electrostatic force to engineer GMPs. **(B)** The BMSCs and PDGF-BB loaded GMs, or “the living GMs” were further developed through incubation with exogenous BMSCs. **(C)** The living GMs were injected into the joint cavity of the DMM rat. The enhanced paracrine activity was achieved by integrating the endogenous and exogenous regeneration mechanisms (Li et al., 2023).

Cartilage lesions and defects caused by trauma, tumors, and inflammation are more common in the clinic. Cartilage is a tissue without blood vessels and nerves (Mow et al., 1992; Ahmed and Hincke, 2010; Gu et al., 2023), and it is extremely difficult to repair itself after injury, which seriously affects the health of patients. At present, cartilage defects can be repaired and reconstructed by cartilage tissue transplantation, chondrocyte or mesenchymal stem cell transplantation, and biomaterial filling. However, these methods have certain limitations, such as limited sources of autologous cartilage and allogeneic cartilage rejection (Haq-Siddiqi et al., 2023). Therefore, repair of cartilage defects and functional reconstruction are still difficult problems in surgical treatment. In recent years, the rapid development of tissue engineering technology has provided new directions for solving the above problems (Ma et al., 2005). Nanofibers loaded with growth factors can combine with seed cells to repair and regenerate cartilage tissue. Kartogenin (KGN) is a newly discovered small molecule compound that has a strong ability to promote cartilage differentiation and can effectively promote the differentiation of mesenchymal stem cells into chondrocytes (Xu et al., 2015). Coaxial electrospun fibers using poly (L-lactic acid-co-caprolactone) and collagen solution as shell fluid and KGN solution as core fluid was fabricated via coaxial electrospinning. KGN encapsulated in the core-shell nanofibers can be sustainedly released for about 2 months. The chondrogenic differentiation of bone marrow mesenchymal stem cells cultured on core-shell nanofibrous scaffold was promoted obviously (Yin et al., 2017). KGN can also be loaded into microspheres via microfluidics to realize the sustained release and eventually repair the cartilage (Wu et al., 2020). An injectable double positively charged functional hydrogel microsphere with “targeting cartilage extracellular matrix”, “cartilage penetration”, and “cellular phagocytosis” can also be designed for matching the structural characteristics of joints and addressing the difficulties of drug delivery in cartilage (Lin et al., 2022). Cartilage repair requires a large supply of cells, therefore microspheres can also be used as injectable cell carriers for cartilage tissue engineering (Li et al., 2023) (Figure 9).

### 4.3 Vessel

In recent years, cardiovascular diseases have become one of the most serious diseases that threaten human health (Tan et al., 2013). When vascular disease reaches the end stage, it is often necessary to use biologically active blood vessels for transplantation or bypass (Ezhilarasu et al., 2019). Due to the limited sources of autologous blood vessel transplantation and the limitations of their own vascular conditions, many of artificial blood vessel replacements are clinically required (Weinberg and Bell, 1986; Jia et al., 2013). In the past, various methods of allogeneic vascular transplantation have been tried, but their long-term effects were poor, including increased thrombosis, vascular degeneration and hyperplasia, and vascular stenosis. The introduction of tissue engineering in recent years have provided new options for this clinical problem. Based on cell biology, material science, and engineering as the basic theory, it can culture and expand seed cells *in vitro* and plant them in biocompatible organisms. A cell–biomaterial complex is formed and implanted in the body to replace tissue defects caused by diseases and trauma, in order to promote the repair and functional reconstruction of the host tissue structure (Wu et al., 2010; Fu et al., 2014). Micro/nano-materials can mimic the microstructure of blood vessels, regulate the microenvironment, and provide biological signals for blood vessel regeneration.

**FIGURE 10:**
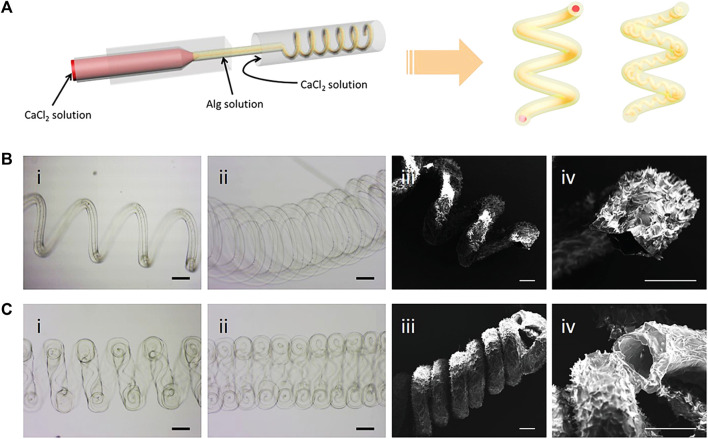
Generation of helical hollow alginate hydrogel microfibers with different channels. **(A)** Schematic illustration of the fabrication of the helical hollow microfibers. **(B, C-i, ii)** Optical microscopy and iii, iv) SEM images of helical hollow microfibers with **(B)** straight and **(C)** helical channels. Scale bars, 200 μm (Jia et al., 2019).

Nanofiber scaffolds are commonly used in vascular tissue engineering. Many studies have shown that it plays an important role in the repair and regeneration of blood vessels (Merkle et al., 2015a). Nanofiber scaffold can simulate the structure of blood vessels. PCL/collagen scaffold prepared via coaxial electrospinning was a suitable scaffold for vascular regeneration. PCL as the core can provide the mechanical property and integrity while collagen as the shell can improve the attachment and proliferation of vascular cells due to its excellent biocompatibility. The collagen shell was crosslinked by genipin and further bound with heparin. The scaffold can support the attached vascular cells to grow and proliferate on its surface, and also allows the infiltration of SMCs into its interior. The heparinized PCL/collagen scaffold with core/shell fiber structure has a promising application in vascular tissue engineering (Duan et al., 2016). Coaxial electrospun PCL/Gelatin-MA fibers which are composed of a PCL core and a functionalized gelatin (GelMA) shell were also proved to be suitable for vascular regeneration (Coimbra et al., 2017). Core-shell PVA/Gelatin electrospun nanofibers promote vascular tissue regeneration due to the potential of altering the proliferation and migration of HUVECs and rat smooth muscle cells (rSMC) (Merkle et al., 2015b). Highly aligned hyaluronan/PLLA nanofibers in core-shell structure were also reported to be an optional vascular graft (Yuan et al., 2016). The hollow microfibers created by microfluidic technology may simulate the structural characteristics of blood vessels. The scaffold is also biocompatible, allowing cells to proliferate and spread within the tube (Jia et al., 2019) (Figure 10).

### 4.4 Skin

Skin trauma, especially large-area full-cortex trauma is still one of the main diseases. The methods of accelerating the healing of the wound surface and reducing the occurrence of scars remain a clinical problem (Chaudhari et al., 2016; Yao et al., 2017). Skin transplantation is the primary method of treating such skin trauma, but patients with large-scale skin defects or skin burns who receive their own skin transplantation will have new wounds on the skin from other areas. The shortage of autologous skin supply is another major factor that limits its application (Wu et al., 2021). Tissue-engineered skin organically combines engineering and life science principles to build skin replacements that improve, maintain, and restore function of the skin, and is expected to solve problems such as insufficient donor skin in repairing skin defects (Dai et al., 2004; Groeber et al., 2011; Li et al., 2023).

The nanofiber scaffold as an important part of skin tissue engineering provides a feasible method for promoting wound healing (Movahedi et al., 2020). Compared with traditional wound dressings, nanofibers have remarkable properties as dermal substitutes, because their structures are very similar to the skin’s primary ECM, and cells can adhere, proliferate, and permeate scaffolds, inducing the regeneration of skin. Lawsone (2-hydroxy-1,4-naphthoquinone) was electrospun in polycaprolactone-gelatin (PCL/Gel) polymers in core-shell architecture to detect the effect of new core-shell nanofibers on wound regeneration. The core-shell nanofibers-encapsulated lawsone prolonged the release of lawsone over a period of 20 days. Moreover, it can promote cell adhesion and proliferation and promote the expression of healing-related genes. *In vivo* experiments further demonstrated that it has a strong promotion effect on wound healing (Adeli-Sardou et al., 2019). Hyaluronic acid-silk fibroin/zinc oxide (ZO) nanofibers can be used in the treatment of burn injuries. ZO as an antibacterial agent can be released sustainedly from the nanofibers. Nanofibers with appropriate ZO loading can effectively inhibit the inflammatory response at the injury site and promote wound healing (Hadisi et al., 2020). Single or multiple growth factors or drugs can be loaded into the core-shell nanofibers in order to achieve the slow release of growth factors and promote wound healing. CTGF is a signaling molecule that has multiple roles in tissue repair and regeneration. CTGF encapsulated electrospun dual porous PLA-PVA core-shell fiber based membranes can be used as excellent wound dressing mats for the treatment of diabetic wounds and other chronic ulcers due to the promotion of cell proliferation, migration and angiogenic potential (Augustine et al., 2019). Biodegradable and biocompatible scaffolds incorporated with multiple epidermal induction factors might serve as the most promising medical devices for skin tissue regeneration. Other studies have shown that aligned nanofiber scaffolds can mimic skin microstructure and induce macrophage M2 polarization (Xie et al., 2022).

The rise of 3D printing technology also provides a new method for the construction of skin tissue engineering scaffolds. Different 3D printing technologies can be used to construct stents with different diameters. 3D printing scaffolds have good biocompatibility and are suitable for cell proliferation and differentiation (Gao et al., 2021). According to the shape and depth of the wound, the 3D printing technology can print out biomaterial suitable for the wound. Scaffolds can also flexibly and accurately load different drugs and growth factors to promote tissue regeneration. Skin tissue can even be printed *in situ* on the wound surface using the layer-by-layer deposition principle (Weng et al., 2021). The integration of electronic skin (e-skin) and tissue has the potential to improve the quality of life for people in the areas of healthcare monitoring and chronic disease treatment. Ga-based alloys can be used to design gluten protein for fabricating e-skin with stretchability, self-healing ability, and biocompatibility. In addition, based on the incorporation of Ga-based alloys, the e-skin obtained has a stronger self-healing ability, and in animal experiments there are no adverse reactions when the e-skin is attached to the skin of rabbits. And in a variety of situations, from large-scale human movement to small strain changes, the e-skin shows a keen ability to sense strain. Therefore, LM-prepared electronic skin has great potential for sensing human movement (Chen et al., 2022).

### 4.5 Annulus fibrosus

Disc herniation is a common disease and one of the main causes of neck and low back pain, which seriously affects the quality of life of patients (Xia et al., 2023). At present, there is no effective means to directly repair damaged AF in clinical practice, and the simple removal of herniated intervertebral disc tissue cannot repair the AF defect at the same time, which has a certain postoperative recurrence rate. Although interbody fusion can treat disc herniation with intervertebral instability and prevent recurrence of disc herniation, loss of intervertebral motion and adjacent segment retreat have become new problems. Therefore, there is an urgent need for AF tissue engineering in clinical practice. The microstructure of AF is a typical angle-ply laminated structure, and this special microstructure also guarantees its good mechanical properties. Aligned nanofibers can simulate the microstructure of AF, which brings great hope for the construction of AF tissue engineering. A novel aligned core-shell nanofibrous scaffold with angle-ply microstructure and co-delivery capacity can achieve burst release of ibuprofen to regulate the microenvironment, and sustained release of TGFβ3 to promote ECM secretion. In addition, it can repair the damaged AF and also realize regeneration of IVD (Han et al., 2022). Micro-nano scaffolds constructed by 3D printing technology can also reproduce the special angle-ply laminated structure of the AF, which is conducive to the growth and proliferation of cells, and can also provide good mechanical support (Bhunia et al., 2021).

## 5 Conclusion and future perspectives

This article reviewed the research progress of biomimetic micro/nano-materials prepared by different methods for the repair and regeneration of different tissues and organs. Common strategies for preparing nanofibers, microspheres and 3D-printing scaffolds were reviewed in order to elucidate the importance of material selection and 3D structure design for tissue repair and regeneration. To date, a large number of studies have shown that using micro/nanomaterials to mimic the microstructure of tissues can promote the repair and regeneration of tissues. Researchers have also modified the surface of scaffolds in multiple ways to adjust the hydrophilicity, adhesion, and mechanical properties of scaffolds. Additionally, studies have shown that these micro- and nano-structured scaffolds can also load drugs, ensuring both the biological activity of drugs and controlled drug release. However, it is not easy to prepare 3D scaffolds with suitable mechanical properties, porosity, surface morphology, degradation rate, and microstructure simulation. The accuracy of existing micro/nanomaterials is limited, and the microstructure of tissues and organs is complex. Therefore, it is still difficult to precisely mimic the microstructure. In addition, the existing micro/nanomaterials focus on the simulation of the microstructure and ignore the simulation of their components, which is another major problem of tissue engineering. The application of micro/nanomaterials is still in its infancy, and there are still many problems to be solved in clinical application such as the way of constructing an ideal cell-nanomaterial interface; preventing immune system, maintaining the survival rate and function of cultured cells for a long time, improving the biocompatibility of nano-materials. It is important to solve the above problems to prepare micro/nano “smart” materials with specific functions to better regulate the biological behaviors such as specific adhesion, proliferation and directional differentiation of seed cells, so as to obtain good biological activity and good biocompatibility and finally apply them to clinic.
